# A Single Dose SARS-CoV-2 Replicon RNA Vaccine Induces Cellular and Humoral Immune Responses in Simian Immunodeficiency Virus Infected and Uninfected Pigtail Macaques

**DOI:** 10.3389/fimmu.2021.800723

**Published:** 2021-12-21

**Authors:** Megan A. O’Connor, Jesse H. Erasmus, Samantha Randall, Jacob Archer, Thomas B. Lewis, Brieann Brown, Megan Fredericks, Skyler Groenier, Naoto Iwayama, Chul Ahrens, William Garrison, Solomon Wangari, Kathryn A. Guerriero, Deborah H. Fuller

**Affiliations:** ^1^ Department of Microbiology, University of Washington, Seattle, WA, United States; ^2^ Washington National Primate Research Center, University of Washington, Seattle, WA, United States; ^3^ HDT Bio, Seattle, WA, United States

**Keywords:** vaccine, nonhuman primate, SIV, COVID-19 vaccine, replicon RNA, SARS-CoV-2

## Abstract

The ongoing COVID-19 vaccine rollout is critical for reducing SARS-CoV-2 infections, hospitalizations, and deaths worldwide. Unfortunately, massive disparities exist in getting vaccines to vulnerable populations, including people living with HIV. Preliminary studies indicate that COVID-19 mRNA vaccines are safe and immunogenic in people living with HIV that are virally suppressed with potent antiretroviral therapy but may be less efficacious in immunocompromised individuals. This raises the concern that COVID-19 vaccines may be less effective in resource poor settings with limited access to antiretroviral therapy. Here, we evaluated the immunogenicity of a single dose COVID-19 replicon RNA vaccine expressing Spike protein (A.1) from SARS-CoV-2 (repRNA-CoV2S) in immunocompromised, SIV infected and immune competent, naïve pigtail macaques. Moderate vaccine-specific cellular Th1 T-cell responses and binding and neutralizing antibodies were induced by repRNA-CoV2S in SIV infected animals and naïve animals. Furthermore, vaccine immunogenicity was elicited even among the animals with the highest SIV viral burden or lowest peripheral CD4 counts prior to immunization. This study provides evidence that a SARS-CoV-2 repRNA vaccine could be employed to induce strong immunity against COVID-19 in HIV infected and other immunocompromised individuals.

## Introduction

Global implementation of vaccines against COVID-19 are necessary to reduce SARS-CoV-2 infections, hospitalizations, and mortality. However COVID-19 vaccine immunogenicity can be lower in immunocompromised individuals (1, 2). Preliminary studies indicate that COVID-19 vaccination is safe and immunogenic in people living with HIV that are virally suppressed on potent combination antiretroviral therapy (cART) but may be less immunogenic in HIV-infected individuals that have limited access to cART and whom have low CD4 counts (< 350 cell/mm^3^) or are immunosuppressed (3–6). Access to cART that is needed to reduce HIV viral burden is limited in resource poor settings and hence untreated HIV infection is a driver of morbidity and mortality in people living with HIV. Furthermore, inequitable distribution of COVID-19 vaccines, especially in low- and middle-income countries, where HIV is endemic, prevents vaccine access by many vulnerable populations. The emergence of SARS-CoV-2 lineages, especially in countries with poor access to COVID-19 vaccines, further jeopardizes achieving complete vaccine coverage. Thus, from a public health perspective it is necessary to study COVID-19 disease and vaccines in immunosuppressed and untreated HIV populations.

Recent reports demonstrate that HIV infection is an independent risk factor for severe COVID-19 and mortality (7). Additionally, people living with HIV have a disproportionate risk of cardiovascular, kidney, and liver disease, cancer, and diabetes, which are co-morbidities associated with increased risk of COVID-19-related hospitalization and death (8–10). Many of the studies evaluating COVID-19 risk in people living with HIV do not capture the >25% of individuals who are untreated (11) and studies are needed to understand specific risks during untreated or advanced HIV.

Previously, we reported that vaccination with a single dose or two doses of a self-amplifying replicon RNA (repRNA) SARS-CoV-2 vaccine, encoding the SARS-CoV-2 (S) spike protein, repRNA-CoV2S, generated robust binding and neutralizing antibody (nAb) responses in mice and nonhuman primates (NHP) (12). A phase I human clinical trial in India with this vaccine is nearing completion and phase I trials in Brazil, Korea, Philippines, China, and the US and a phase II/III trial in India are pending (13–15). Decreased vaccine immunogenicity to infectious diseases remains a significant issue for people living with HIV, even among those with access to potent cART (16). To overcome these limitations, people living with HIV often require higher vaccine doses, additional booster doses or co-delivery with potent adjuvants to develop sufficient levels of immunity (17). Current COVID-19 mRNA vaccines have >90% efficacy at preventing COVID-19 and hospitalization, however in individuals who are immunocompromised, vaccine efficacy is reported to be ~20-30% lower (18–20). Unlike “conventional” mRNA vaccines, repRNA vaccines trigger innate pathways, activate Toll-like receptors and promote cross-priming that can contribute to eliciting more robust immune responses (21). Consequently, this vaccine platform is self-adjuvating and ideal for inducing immune responses in immunosuppressed individuals. Here, we used the pre-clinical simian immunodeficiency virus (SIV) NHP model for human HIV infection to investigate repRNA-CoV2S vaccine immunogenicity in SIV-infected pigtail macaques during analytic antiretroviral treatment interruption.

## Materials and Methods

### Pigtail Macaques and Sample Collection

A total of 15 male pigtail macaques (aged 2.1-5.1 years, 3.6-9.6 kg) were used. **Supplemental Table 1** details animal characteristics, including MHC haplotypes. Nine animals were experimentally infected with SIV (described in detail below) and 6 animals remained naïve. All animals received a single immunization (5 or 25 μg) of repRNA-CoV2S and were then studied for 6 weeks. As previously described (12), all animals were housed at the Washington National Primate Research Center (WaNPRC), an accredited facility the American Association for the Accreditation of Laboratory Animal Care International (AAALAC). All animal procedures were approved by the University of Washington’s Institutional Animal Care and Use Committee (IACUC) (IACUC #4266-14). Animals were sedated with an intramuscular injection (10 mg/kg) of ketamine (Ketaset^®^; Henry Schein) for blood collections. Serum, plasma, and peripheral blood mononuclear cells (PBMCs) were isolated from whole blood as previously described (12). The animal’s general health was observed daily and full physical exams were conducted at each experimental timepoint, as previously described (12). Only SIV-infected animals were euthanized at the study endpoint at 6 weeks post-immunization under deep anesthesia, in accordance with the 2007 American Veterinary Medical Association Guidelines on Euthanasia, by administration of Euthasol^®^ (Virbac Corp., Houston, TX).

### SIV Infection and cART Regimen

Nine pigtail macaques were infected intravenously with 10,000 infectious units (I.U.) of SIVmac239M (22) (gift from Dr. Brandon Keele, AIDS and Cancer Virus Program, Frederick National Laboratory for Cancer Research). Animals were given a combination antiretroviral therapy (cART) regimen for 26 weeks starting at 4 weeks post-SIV infection consisting of 1mL/kg of a mixture of the following antiretrovirals administered daily by intramuscular (i.m.) injection: 2.5 mg/kg Dolutegravir (DTG) (Gilead Sciences, Foster City, CA), 5.1 mg/kg tenofovir disoproxil fumarate (TDF) (ViiV Healthcare, Research Triangle, NC), and 30 mg/kg emtricitabine (FTC) (Gilead Sciences, Foster City, CA) in 15% Kleptose in water (Roquette, Geneva, IL). These SIV-infected animals were previously enrolled in another study where following SIV infection and during cART, they received 3 doses of an experimental hepatitis B virus (HBV) vaccine consisting of a combination of CD180 targeted DNA and recombinant protein vaccines comprised of HBV core and surface antigens (n=4) (23) or a commercial Engerix-B vaccine (GlaxoSmithKline, Research Triangle Park, NC) (n=5) (**Supplemental Table 1**). Analytic treatment interruption (ATI) was performed and then in response to the COVID-19 pandemic, these animals were re-enrolled in this study after viral loads had rebounded and were immunized with the repRNA-CoV2S vaccine at 8 weeks post-ATI. Animals were then euthanized 6 weeks after the single repRNA-CoV2S dose consistent with the original experimental endpoint associated with the HBV vaccine study. Animals receiving each HBV vaccine were evenly distributed between the repRNA-CoV2S vaccine groups (**Supplemental Table 1**).

### RepRNA-CoV2S Immunization

Macaques were immunized with an alphavirus-derived replicon RNA SARS-CoV-2 vaccine encoding the full-length A.1 lineage (GenBank: MN908947.3) SARS-CoV-2 Spike (S) protein (repRNA-CoV2S). This repRNA vaccine is comprised of codon optimized DNA gene sequences fused to a c-terminal v5 epitope tag and cloned into a plasmid vector encoding untranslated regions and a non-structural open reading frame of Venezuelan equine encephalitis virus strain TC-83 (12). To protect repRNA from degradation and enhance stability, repRNA was co-formulated with a Lipid InOrganic Nanoparticle (LION) emulsion consisting of inorganic superparamagnetic iron oxide (SPIO) nanoparticles and squalene core (HDT Bio, Seattle, WA). The stability, safety, and immunogenicity of repRNA-CoV2S was described previously in detail (12). The vaccine was prepared as previously described (12) and delivered by i.m. injection into the quadriceps and deltoid muscles. Animals received one of the following repRNA-CoV2S vaccine doses: 1) 25 μg delivered over 5 i.m. sites (n=3 SIV+, n=3 naïve), 2) 5 μg delivered over 5 i.m. sites (n=3 SIV+, n=3 naïve), or 3) 5 μg delivered over 1 i.m. site [single site (SS)] (n=3, SIV+), as shown in **Supplemental Table 1**. All injection sites were monitored post-immunization for any signs of local reactogenicity.

### Simian AIDS Measurements

Animals were monitored for Simian AIDS as defined by the WaNPRC guidelines (24). Briefly, weights, complete blood counts, and peripheral blood CD4 counts were monitored at each specimen collection timepoint. SIV plasma viremia was evaluated by quantitative real time reverse transcription polymerase chain reaction (RT-PCR) by the Virology and Immunology Core at the WaNPRC, as previously described (24). Peripheral CD4 counts were determined from complete blood counts (12) (University of Washington Department of Laboratory Medicine) using flow cytometry based methods by the Virology and Immunology Core at the WaNPRC, as previously described (25).

### IL-4/IFN-γ Enzyme-Linked Immunospot Assay (ELISPOT)

Antigen-specific PBMCs secreting IL-4 or IFN-γ were detected using a Human IFN-γ/IL-4 Double-Color ELISPOT (ImmunoSpot, Shaker Heights, Cleveland, OH), per manufacturer’s protocol. Briefly, cryopreserved PBMC cells were thawed, and 1-3 x 10^5^ cells were stimulated for 48 hours with 11 SARS-CoV-2 Spike peptide pools (17- or 18-mers with 11 amino acid overlap) (Genscript, Piscataway, NJ) at a concentration of 1μg/mL per peptide. DMSO and Concanavalin A (ThermoFisher, Waltham, MA), were used as negative and positive controls, respectively, as previously described (12). Spots were counted on an Immunospot Analyzer with CTL Immunospot Profession Software (Cellular Technology Ltd., Shaker Heights, Cleveland, OH). Spot forming cells (SFC) in peptide stimulated wells were computed following subtraction of SFCs detected in DMSO stimulated control wells and were considered positive if the number of SFC was > 1 spot per 1 x 10^5^ plated cells.

### SARS-CoV-2 Binding Antibody

Antigen-specific IgG responses were detected in the sera by enzyme linked immunosorbent assay (ELISA) using a recombinant SARS-CoV-2 S protein as the capture (26), and performed as previously described (12). Briefly, ELISA plates were coated with 1 μg/mL antigen and serially diluted serum samples were added and detected *via* anti-monkey IgG-HRP (Southern Biotech, Birmingham, AL) and then developed using a TMB substrate (Fisher Scientific, Waltham, MA). Plates were analyzed at 405nm (ELX808, Bio-Tek Instruments Inc) and serum concentrations were determined from a standard curve, as previously described (12).

### SARS-CoV-2 Neutralizing Antibody

Eighty percent (80%) plaque-reduction neutralizing antibody titers (PRNT80) against the SARS-CoV2/WA/2020 (BEI Resources, Manassas, VA) were determined in two-fold serial diluted heat inactivated serum, as previously described (12). Briefly, serum and virus were incubated and plated onto Vero E6-TMPRSS2 cells (gift from Dr. Michael Gale, University of Washington) and incubated for 1hr at 37°C. Following adsorption, wells were overlaid with 0.2% agarose in DMEM and incubated for 2 days at 37°C. The overlay was then removed, washed, fixed using 10% formaldehyde (Sigma-Aldrich, St. Louis, MO), and stained with 1% crystal violet (Sigma-Aldrich, St. Louis, MO) in 20% EtOH (Fisher Scientific, Waltham, MA). Plaques were enumerated and percent neutralization was calculated relative to the virus-only control.

### Quantification of Gut Barrier Dysfunction

Plasma quantification by ELISA of human soluble CD14 (sCD14), human C-reactive protein (CRP), human fatty acid binding protein 2 (FABP2) (Fisher Scientific, Waltham, MA) or human LPS binding protein (LBP) (Biometec, Germany) was performed per the manufacturer’s instruction. Plasma was diluted as follows: 1:200 (sCD14), 1:2 (FABP2), 1:1,000 (CRP), or 1:3 (LBP). Results were analyzed using a four-parameter logistic (4-PL) function for fitting standard curves using Prism version 8.4.3 (GraphPad).

### Statistical Analyses

Statistical analyses were conducted in Prism v.8.4.3 (Graphpad, San Diego, CA, USA). Comparisons between groups were determined by Mann–Whitney test. Correlations were evaluated by Spearman Rank test. Statistical significance was considered achieved when the *p*-values were < 0.05.

## Results

### Experimental Design of repRNA-CoV2S Vaccination in Naïve and Immunocompromised, SIV-Infected Pigtail Macaques

**Figure 1:**
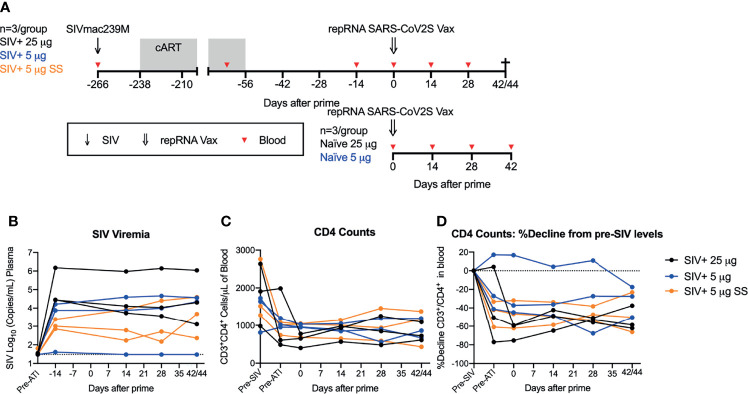
repRNA-CoV2S vaccination in SIV-infected, immunosuppressed pigtail macaques. **(A)** Pigtail macaques (n=9) were infected with SIVmac239M and put on 26 weeks of cART starting 4 weeks after infection. During cART animals received an HBV DNA and protein vaccine regimen, comprised of HBV core and surface antigens and anti-CD180, prior to enrollment in the COVID-19 vaccine study. cART was stopped 8 weeks prior to receiving an intramuscular immunization of repRNA-CoV2S (RepRNA vax) (25 μg (n=3, black circles); 5 μg [n=6 (blue and orange circles)] delivered over 5 sites (black and blue circles) or single site (SS) (orange circles). repRNA-CoV2S was also delivered to naïve control animals [25 μg (n=3); 5 μg (n=3)]. Blood was collected at baseline and days 10, 14, 28, and 42/44 DPI. SIV+ animals underwent an experimental necropsy associated with the HBV-vaccine protocol on days 42/44 post-vaccination. **(B)** Plasma levels of SIV viral RNA levels were measured by RT-PCR, the dotted line the limit of detection (30 copies/mL of plasma). **(C)** Peripheral blood CD3+CD4+ T-cell counts were quantified from the complete blood count (CBC) following flow cytometry analysis. **(D)** Decline in peripheral blood CD3+CD4+ T-cell counts were calculated relative to the percent of pre-SIV infection levels. The dotted line indicates pre-SIV infection levels.

Previously, we demonstrated robust immunogenicity with one or two doses of the repRNA-CoV2S vaccine in naïve pigtail macaques (PTM) (Macaca nemestrina) (12). Given the ability of repRNA vaccines to induce robust innate immune responses (27), we hypothesized that repRNA-CoV2S would also be able to induce strong antibody and T cell responses in immunocompromised SIV-infected NHPs. To determine if this vaccine could induce strong immune responses in a non-human primate model of HIV/AIDS that closely resembles immunocompromised people living with HIV, we immunized SIV infected PTMs (N=9) 8 weeks after cART was withdrawn and when viral loads had rebounded. The PTMs were previously infected intravenously with SIVmac239M, a barcoded virus allowing for the distinction of almost 10,000 isogenic clones (22), and then put on cART for a period of 6 months starting 4 weeks after infection (**Figure 1A**). During the cART period, and as part of another experiment, the PTMs received an experimental hepatitis B virus (HBV) vaccine (**Supplemental Table 1**) and then underwent an analytic treatment interruption (ATI) that resulted in rapid rebound of SIV viral replication.

Vaccine immunogenicity in the nine SIV infected PTMs was compared to six naïve PTMs given the same repRNA-CoV2S vaccine. Previously we evaluated administration of repRNA-CoV2S at 50 μg over one injection site or 250 μg over five injection sites (12). To support our clinical development activities with the repRNA-CoVS vaccine platform, we employed a dose de-escalation study exploring the immunogenicity at two lower vaccine doses (25 μg or 5 μg) in naïve PTMS. Here, we did parallel vaccine immunogenicity studies in SIV-infected pigtail macaques at these lower doses. To continue to bridge our pre-clinical vaccine model for use in the clinic, we also tested administration of the 5 μg vaccine in the SIV+ cohort delivered over five (n=3) or a single (SS, n=3) injection site(s) (**Supplemental Table 1**). Altogether, the vaccinated experimental groups were as follows (n=3/group): SIV+ 25 μg, SIV+ 5 μg, SIV+ 5 μg SS, naïve 25 μg, naïve 5 μg (**Figure 1A** and **Supplemental Table 1**). All SIV-infected animals were immunized with a single dose of the repRNA-CoV2S vaccine at 8 weeks after cART withdrawal. At the time of immunization, the median ± interquartile range level of SIV viral RNA in the plasma was 3.9 ± 4.6 log_10_ copies/mL, with a single animal (Z15182, SIV+ 5 μg) exhibiting SIV viral control at or below the limit of detection (**Figure 1B** and **Supplemental Table 2**). In addition, frequencies of peripheral blood CD4 counts ranged from 402 to 1046 cells/μL of blood (**Supplemental Table 1**) and corresponded to declines of up to 75.3% relative to baseline levels measured prior to SIV infection (**Figures 1C, D** and **Supplemental Table 1**). A single animal (Z14302, SIV+ 5 μg) did not have depletion of peripheral CD4 T-cells at the time of vaccination (**Figure 1D** and **Supplemental Table 1**). Evidence of gut barrier dysfunction was minimal at the time of vaccination, with only 2 animals displaying elevated plasma levels of lipopolysaccharide binding protein (LBP) compared to pre-infection levels (**Supplemental Figure 2**). Thus, at 8 weeks post-cART withdrawal when the vaccine was administered, 8/9 SIV-infected animals exhibited viral rebound and 8/9 exhibited a range in immunosuppression as indicated by declines in peripheral CD4 T cell counts. Animals were monitored for 6 weeks, and blood was collected prior to vaccination and at 14, 28, and 42 or 44 days post immunization (DPI) to evaluate vaccine safety and immunogenicity including analysis of cellular and humoral immune responses. Vaccine follow-up of 6 weeks (42/44 days) in the SIV-infected animals was limited by an experimental endpoint associated with the original HBV vaccine study (see *Methods*). Consistent with our previous study in uninfected animals (12), no acute adverse reactions were observed in any of the animals following immunization (**Supplemental Figure 1**).

### Low Doses of repRNA-CoV2S Induce Strong Antibody and Modest T Cell Responses in SIV-Infected Pigtail Macaques

**Figure 2:**
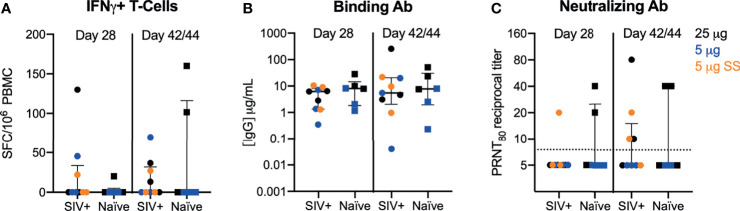
repRNA-CoV2S vaccination in SIV-infected pigtail macaques induces robust cellular and humoral immune responses. PBMCs and sera were isolated from blood at 28 and 42/44 DPI for vaccine immunogenicity. **(B)** The magnitude of IFN-γ-producing T-cells were measured in PBMCs following 48-hour stimulation with 11 peptide pools encompassing the SARS-CoV-2 spike (S) protein using a dual IFN-γ/IL-4 ELISpot assay. The number of spot forming cells (SFCs) per 10^6^ PBMC are shown. IL-4-producing T cell responses were low or undetectable in all animals post-vaccination and are not shown (see **Supplementary Figure 4**). **(C)** Serum anti-S IgG enzyme linked immunosorbent assays were measured by ELISA and **(D)** 80% plaque-reduction neutralizing antibody titers (PRNT80) against the SARS-CoV2/WA/2020 isolate were measured by plaque reduction neutralization test **(B–D)** Medians with interquartile ranges are shown. No significance was found by Mann-Whitney test between SIV+ and naïve animals at either timepoint.

Cellular and humoral immune responses to the vaccine were evaluated at 28 and 42/44 DPI. Within the SIV cohort, similar levels of antibody and T cell responses were observed for both the 5 and 25 ug dose levels regardless of whether the dose was administered over a single or five sites; therefore, data were combined for comparative analyses (**Supplemental Figure 3 **and** Supplemental Table 2**). In our previous studies, we measured IFN-γ T cell responses by ELISPOT and polyfunctional T cell responses expressing IFN-γ, IL-2, IL-4, IL-17A, TNF-α, MIP-1 β, and/or Granzyme B/CD107a by intracellular cytokine staining (ICS). Only mild/moderate T-cell responses elicited by repRNA-CoV2S were detected and T cell responses were almost exclusively IFN-γ secreting cells (12). Therefore, in this study, we focused on IFN-γ and IL-4 T cell responses measured by ELISPOT in our analysis to investigate the effects of SIV infection on Th1 and Th2 responses. Modest frequencies of IFN-γ (Th1) producing T-cells were induced after a single dose in SIV-infected and naïve PTMs (**Figure 2A**). In addition, the magnitude of the IFN-γ T-cell response in the SIV-infected macaques were consistent with levels we previously observed following immunization of naïve PTMs with higher doses (50-250 μg) of the same vaccine (12). Overall, T-cell responses were detected in 30-45% of all animals (4/9 SIV+ and 2/6 naïve) at 42/44 DPI. Vaccine induced Th2 (IL-4) T-cells were detected at very low levels only in a single SIV-infected animal at 28 DPI and in none of the naïve animals (**Supplemental Figure 3**). The Th1 T-cell response was primarily directed against the Spike 2 subunit (**Supplemental Figure 4**) in both SIV+ and naïve animals with only 2 PTM (1 SIV+ and 1 naïve) exhibiting a dominant T-cell response against the Spike 1 subunit that contains the receptor binding domain (RBD). These results are in contrast to our previous study where we showed that 2 to 10-fold higher doses (50-250 μg) of the repRNA-CoV2S vaccine in naïve PTMs induced a dominant T cell response against the S1/RBD region (12). These data suggest that the dose of repRNA-CoV2S may influence the specificity or repertoire of T-cell responses.

We next evaluated humoral responses in the sera. Robust binding antibodies (bAb) against the S protein were detected in all animals (**Figure 2B**). Concentrations of anti-Spike IgG of 0.35 to 28 μg/mL observed at day 28 in this study were comparable to IgG concentrations (0.5 to 45 μg/mL) previously reported after a single, higher dose of 50 or 250 μg repRNA-CoV2S (12). Despite seroconversion in all PTM, variable levels of antibody virus neutralizing titers were detected 28-42/44 DPI (**Figure 2C**). At 28 DPI, 1/9 SIV-infected and 2/6 naïve animals had detectable neutralizing antibody (nAb) responses and by 42/44 DPI, nAb were detected in 4/9 and 2/6 in SIV-infected and naïve PTM, respectively (**Figure 2C**). At 42/44 DPI, nAb responses were detected in 66% (4/6) of PTMs receiving the 25 μg dose versus 22% (2/9) of PTMs receiving the 5 μg dose, irrespective of SIV status (**Figure 2C**). Overall, a better responder rate and significantly higher levels of nAb responses were detected at the 25 μg versus the 5 μg dose (**Supplementary Table 2**). Furthermore, nAb levels induced with the 25 μg dose in both naïve and SIV-infected PTMs were comparable with levels induced using higher doses of the vaccine in our previous study in naïve PTMs (12), but the response rate was lower in all animals receiving doses <25 μg. These data indicate that the dose of repRNA-SARS-CoV2S needed to induce optimum nAb responses against SARS-CoV-2 is >5 μg.

### The Immunogenicity of the repRNA-CoV2S Vaccine Is Not Impacted by the Level of SIV-Induced Immunosuppression

**Figure 3:**
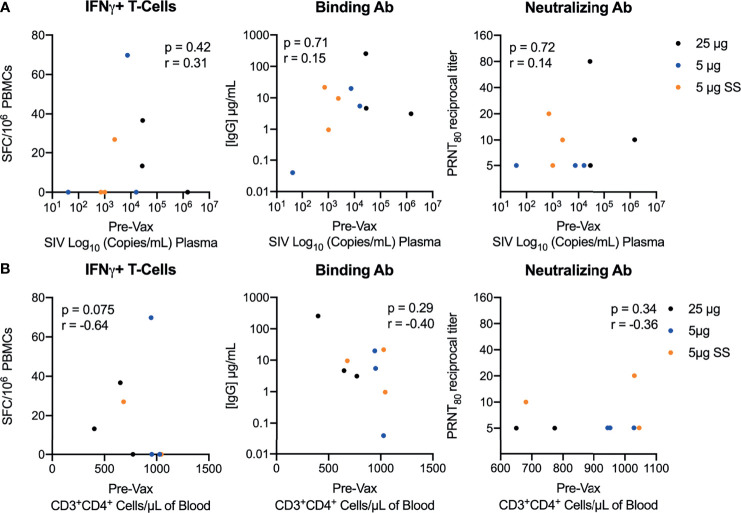
Vaccination with repRNA-SARS-CoV2S induces strong immunity despite SIV-induced immunosuppression. Correlations between pre-vaccination levels of **(A)** SIV viremia or **(B)** peripheral CD4 counts versus T-cell magnitude (left panel) and binding (middle panel) or neutralizing (right panel) antibody responses 42/44 DPI. Spearman’s rank correlation is shown, with p-values ≤ 0.05 considered significant.

At the time of immunization, the SIV-infected macaques exhibited varying levels of SIV disease progression, with pre-vaccination viral loads ranging from 1.61 to 6.17 log_10_ SIV RNA copies/mL and CD4 counts ranging from 402 to >1000 cells/μL of blood (**Supplemental Table 1**). To determine if the immunogenicity of the repRNA-CoV2S vaccine was impacted by SIV disease phenotypes, bAb, nAb and frequencies of Th1 T-cell responses measured 42/44 days post-vaccination were compared to viral loads and CD4 counts present just prior to vaccination. Overall, there was no significant correlation between the magnitude of bAb, nAb or T cell responses and viral loads, frequencies of peripheral CD4 counts (**Figure 3**), relative decline in CD4 counts compared to pre-SIV baseline levels. (**Supplemental Figure 5**), nor with plasma levels of markers of gut barrier dysfunction (**Supplemental Figure 6**). Collectively, these data demonstrate that the immunogenicity of the repRNA-CoV2S was not impacted by SIV-induced immunosuppression.

## Discussion

Here, we demonstrate a SARS-CoV-2 alphavirus-derived replicon RNA vaccine formulated with a novel Lipid InOrganic Nanoparticle (LION) emulsion (repRNA-CoV2S) induced robust cellular and humoral immune responses after a single immunization in immunocompromised SIV-infected animals as a highly relevant model of untreated HIV infection in humans. Collectively, this pilot, proof-of-concept study provides strong evidence that a SARS-CoV-2 repRNA vaccine could be employed to induce immunity against COVID-19 in HIV infected and other immunocompromised individuals.

Preliminary studies indicate that mRNA or adenovirus-vectored COVID-19 vaccination is safe and immunogenic in treated and virally suppressed people living with HIV (3, 5, 6). In addition, antibody and T-cell responses following natural SARS-CoV-2 infection in virally suppressed HIV infected individuals on antiretroviral drug therapy do not appear to be dampened when compared to HIV uninfected individuals (28). However, there is evidence that responsiveness to COVID-19 vaccination and the durability of vaccine-induced immunity may be dampened in non-HIV-associated immunocompromised individuals (1, 2, 18, 19) and individuals living with HIV who are non-virally suppressed and have low CD4 counts (3–5). In addition, there is growing evidence that there may be a role of HIV-associated immune impairment, in particular lower CD4^+^ T-cell counts, with susceptibility to SARS-CoV-2 infection or COVID-19 severity (29, 30). Our data suggests that self-amplifying repRNA vaccines may enable induction of protective immunity in these vulnerable, immunocompromised populations. Importantly, unlike current mRNA COVID-19 vaccines, this vaccine platform utilizes an ad-mix formulation that can be manufactured independently of the RNA component and combined with the RNA vaccine immediately prior to immunization. The LION formulation used to deliver our repRNA vaccine can be stored long-term at 4°C or room temperature (12). The stability of this vaccine platform makes it more amenable than currently licensed mRNA vaccines for rapid distribution in low income and resource poor settings where it can be locally stockpiled and readily available for co-formulation with an updated RNA vaccine customized to each region depending on local virus epidemiology and in response to emerging variants of concern and future pandemics. Since the RNA vaccine does not need to be encapsulated into lipid nanoparticles under a regulated manufacturing process, formulating variant-specific vaccines with LION is more flexible and can be more rapidly customized.

Many nonhuman primate species including rhesus macaques (Macaca mulatta), cynomolgus macaques (Macaca fascicularis), African green monkeys (Chlorocebus aethiops) and pigtail macaques reported here (Macaca nemestrina), have been critical for investigating COVID-19 disease and pre-clinical testing of COVID-19 vaccines (31, 32). Recent findings from Melton et al., demonstrate moderate SARS-CoV-2 disease in pigtail macaques (33), as compared to the more mild disease observed in rhesus macaques, but with similar levels of viral load in respiratory mucosa between the two species (34). There are several unique features of the pigtail macaque model of HIV infection that are worth noting. SIV infection in pigtail macaques results in a more rapid progression to AIDS-defining events than rhesus macaques (35, 36). The increased disease progression in the pigtail is not due to higher levels of SIV replication, but rather is because of a higher propensity for decreased gut barrier function and increased immune activation (35, 37). Furthermore, studies have demonstrated the pigtail macaque as a model for studying cardiovascular disease and chronic obstructive pulmonary disease (COPD) (38, 39) and thus could be valuable for studying SARS-CoV-2 co-morbidities. The elderly are more vulnerable to severe COVID-19 and death and aged nonhuman primates are useful for recapitulating aspects of severe COVID-19, including acute respiratory distress syndrome (ARDS) and pneumonia (40, 41), but the scarcity of this resource limits its widespread use in COVID-19 research. Additional studies are needed to determine if repRNA-2S retains its immunogenicity in older naïve and SIV-infected macaques.

As a pilot, there are limitations to this study, including the short duration of post-immunization follow-up (6 weeks), the use of a prime only immunization, delivery of a subthreshold vaccine dose (5 μg) that resulted in suboptimal immune responses even in naïve animals, and use of only male animals. Neutralizing antibody responses across the SIV and naïve groups were detected in 30-45% of animals after 6 weeks. This is in contrast to our previous results in animals given a 50 or 250 μg dose (12), suggesting that a longer period of time or a booster immunization may be needed to generate maximum nAb responses at lower vaccine doses (< 25 μg). Despite these limitations, the ability of this vaccine to induce immune responses in fully immune competent uninfected and SIV+/CD4-depleted macaques provides evidence that the repRNA-CoV2S/LION vaccine delivery platform can induce immunity even in the context of immunosuppression. Further studies are needed to directly compare the repRNA/LION to a standard mRNA/lipid nanoparticle vaccine formulation to determine if the ability of the repRNA/LION vaccine to induce robust immune responses in SIV+ immunocompromised animals is unique.

Recent studies suggest T-cells may not be required for generation of anamnestic clearance of SARS-CoV-2 re-infection (42). Thus, production of robust, long-lived humoral immunity following COVID-19 vaccination even in the setting of HIV-induced CD4 depletion and immune exhaustion may be possible. Vaccine seroprotection in people living with HIV can be shorter than in uninfected individuals (43), but there is evidence that durable humoral immunity is detected >6 months after SARS-CoV-2 infection irrespective of HIV status (44). It is possible that HIV/SIV-induced immune dysfunction, especially in individuals with untreated or advanced HIV (45), may negatively impact the durability of repRNA-CoV2S vaccine immune responses and further studies are needed to investigate this. We previously found that the repRNA vaccine platform drives a strong humoral response, but mild cellular immune responses in naïve macaques (12). The ability of the repRNA vaccine to induce robust antibody even in the presence of modest or low T cell responses may be beneficial in individuals with T-cell deficiencies or immune dysfunction. In support of this possibility, the repRNA vaccine induced robust humoral immune responses in animals with low to moderate peripheral CD4+ T-cell counts of 400-750 cells/μL of blood, but further evaluation of COVID-19 vaccines in a setting of severe CD4 depletion (< 250 cells/μL) is needed.

In addition, further studies are needed to determine the impact of HIV infection, in particular, T- and B-cell dysfunction, on the induction of neutralizing and binding Ab responses and on SARS-CoV-2 viral evolution and viral pathogenesis. In immunocompromised individuals, prolonged and persistent SARS-CoV-2 infection could provide fertile ground for viral evolution of more virulent strains (46, 47), that when transmitted could contribute to “breakthrough infections” in vaccinated individuals (45). In conclusion, the repRNA/LION vaccine platform is a viable next generation COVID-19 vaccine to induce protective levels of antibody responses in immunocompromised individuals and may be an ideal COVID-19 vaccine candidate for distribution in resource limited settings.

## Data Availability Statement

The raw data supporting the conclusions of this article will be made available by the authors, without undue reservation.

## Ethics Statement

The animal study was reviewed and approved by University of Washington’s Institutional Animal Care and Use Committee (IACUC) (IACUC #4266-14).

## Author Contributions

Conceptualization: MO, JE, and DF. Methodology: MO, JE, SR, JA, TL, BB, MF, and SG. Formal analysis: JE, SR, JA, TL, BB, MF, and SG. Writing—original draft preparation: MO and DF. Writing—review and editing: MO’C, JE, SR, JA, TL, BB, MF, and DF. Animal studies and veterinary care: NI, CA, SW, WG, and KG. Visualization: MO. Supervision: MO, JE, and DF. Funding acquisition: JE and DF. All authors contributed to the article and approved the submitted version.

## Funding

This work was supported by the National Institute of Health (NIH) grant numbers R56-AI141494 (DF/Clark), P51-OD010425 (PI-Sullivan, DF Co-I, MO’C Co-I), ﻿NIH/NIAID Centers of Excellence for Influenza Research and Surveillance contract 27220140006C (Erasmus). MO’C is also supported by NIH grant K01-MH123258.

## Conflict of Interest

JE, JA, and DF have equity interest in HDT Bio. JE is a consultant for InBios. DF is a consultant for Gerson Lehrman Group, Orlance, Abacus Bioscience, Neoleukin Therapeutics. JE is a co-inventor on U.S. patent application no. 62/993,307 “Compositions and methods for delivery of RNA” pertaining to the LION formulation.

The remaining authors declare that the research was conducted in the absence of any commercial or financial relationships that could be construed as a potential conflict of interest.

## Publisher’s Note

All claims expressed in this article are solely those of the authors and do not necessarily represent those of their affiliated organizations, or those of the publisher, the editors and the reviewers. Any product that may be evaluated in this article, or claim that may be made by its manufacturer, is not guaranteed or endorsed by the publisher.
