# Supplementary material for: A Single Dose SARS-CoV-2 Replicon RNA Vaccine Induces Cellular and Humoral Immune Responses in Simian Immunodeficiency Virus Infected and Uninfected Pigtail Macaques
Source: Front Immunol. 2021 Dec 21;12:800723. doi: 10.3389/fimmu.2021.800723 (PMC8724308; doi:10.3389/fimmu.2021.800723)
Supplement: Supplementary file 1 [file DataSheet_1.docx]

Supplementary Material

# Supplementary Figures and Tables

## Supplementary Figures

##
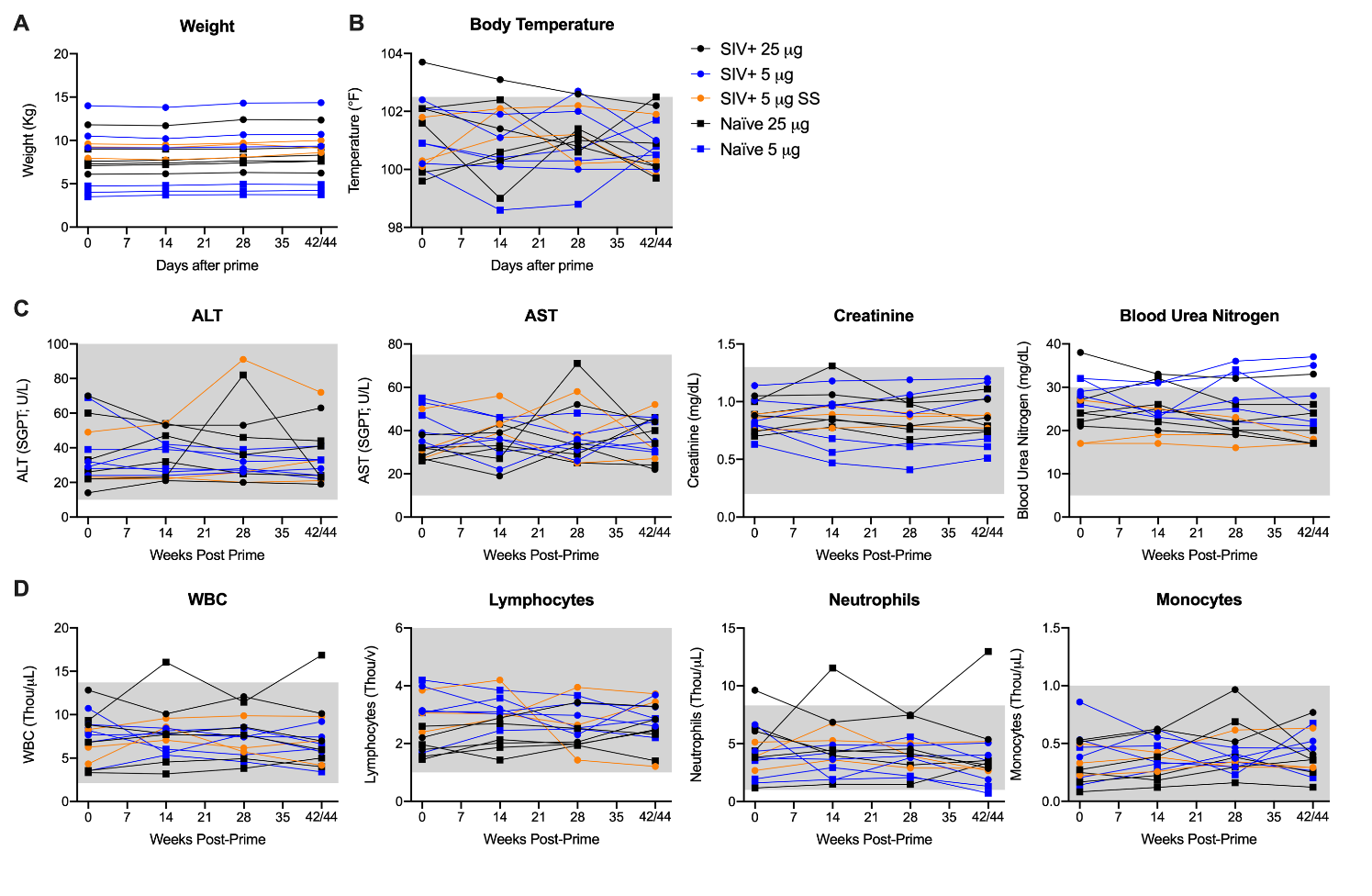
Supplementary Figure 1. No adverse reactions to repRNA-CoV2S in pigtail macaques. (A) Body weight. (B) Rectal body temperature in Fahrenheit. (C) Serum chemistries. (D) Blood complete blood counts (CBC). (A-D) Grey shaded areas indicate normal ranges for pigtail macaques.

**Supplementary Figure 2**. Measurements of gut barrier dysfunction. Markers of microbial translocation were measured in the plasma by ELISA: (A) soluble CD14 (sCD14). (B) C-reactive protein (CRP). (C) LPS binding protein (LBP). (D) Fatty acid binding protein 2 (FABP2). (A,B,D) Dotted lines indicate lower limits of detection for each assay.

**Supplementary Figure 3.** repRNA-CoV2S immunogenicity by vaccine group. (A) Magnitude of IFN-γ T-cell analysis responses measured in PBMCs following 48-hour stimulation with 11 peptide pools encompassing the SARS-CoV-2 spike (S) protein evaluated by dual IFN-γ/IL-4 ELISpot assay. Number of spot forming cells (SFCs) are indicated. (B) Serum anti-S IgG enzyme linked immunosorbent assays (ELISAs) and (C) 80% plaque-reduction neutralizing antibody titers (PRNT80) against the SARS-CoV2/WA/2020 isolate were. (A-C) Medians with interquartile ranges are shown. No significant differences between groups, as determined by Mann–Whitney test.

** Supplementary Figure 4.** Magnitude and breadth of T-cell response. SIV+ or naïve pigtail macaques were vaccinated with a single dose of repRNA-CoV2S. PBMCs were isolated from blood pre-vaccination and on days 28 and 42/44 DPI for T-cell analysis. Shown are magnitudes of (A) IFN-γ and (B) IL-4 T cell responses measured in PBMCs by ELISpot assay following 48-hour stimulation with 11 peptide pools encompassing the full-length spike (S). Shown are the cumulative responses against different peptide pools within the S1 (red shades), S2 (blue shades) and RBD regions (red, P3-P5) of the Spike protein. Data are presented as spot forming cells (SFCs) per 1 million PBMCs.

** Supplementary Figure 5.** Relationship between vaccine immunogenicity and peripheral CD4 decline. Correlations between percent decline in peripheral CD4 counts relative to pre-SIV baseline and magnitude of SARS-CoV-2 Spike-specific IFN-γ T-cell responses measured by ELISPOT (left panel), binding antibody responses measured by ELISA (middle panel) or neutralizing antibody responses measured by PRNT_80_ (right panel) at 42/44 days following a single immunization with repRNA-CoV2S. Spearman’s rank correlation is shown, with p-values ≤ 0.05 considered significant.

**Supplementary Figure 6.** Relationship between vaccine immunogenicity and measurements of gut barrier dysfunction. Correlations between plasma concentrations of (A) sCD14, (B) LBP, and (C) FABP2 prior to vaccination and magnitude of SARS-CoV-2 Spike-specific IFN-γ T-cell responses measured by ELISPOT (left panel), binding antibody responses measured by ELISA (middle panel) or neutralizing antibody responses measured by PRNT (right panel) at 42/44 days following a single immunization with repRNA-CoV2S. Spearman’s rank correlation is shown, with p-values ≤ 0.05 considered significant

## 1.2 Supplementary Tables

| Supplemental Table 1. Animal characteristics, treatment history and repRNA-CoV2S vaccine dosing regimen | | | | | | | | | | | | |
| --- | --- | --- | --- | --- | --- | --- | --- | --- | --- | --- | --- | --- |
|  | | | | | repRNA-SARS-CoV2S vaccine | | SIV parameters at the time of immunization | | | MHC | |  |
| Animal ID | Group | Sex | Age (yrs) | Weight (Kg) | Dose  (μg) | i.m. Sites | Plasma SIV RNA copies/mL (Log_10_) | #CD3^+^CD4^+^ cells/mL of blood | CD3^+^CD4^+^ cells/mL of blood (% relative to pre-SIV) | Haplotype A | Haplotype B | HBV Vaccine^a^ |
| Z16041 | SIV+ | M | 3.4 | 5.1 | 25 | 5 | 6.17 | 773 | 40.5 | Mane-A019 Mane-A031 | Mane-B052 Mane-B069 | DNA +Protein |
| Z15329 | SIV+ | M | 3.8 | 5.3 | 25 | 5 | 4.44 | 402 | 41.0 | Mane-A019 Mane-A032 | Mane-B028 Mane-B052 | DNA +Protein |
| Z14362 | SIV+ | M | 4.7 | 8.8 | 25 | 5 | 4.45 | 650 | 24.7 | Mane-A006 Mane-A032 | Mane-B118 | Engerix-B |
| Z15291 | SIV+ | M | 3.9 | 8.4 | 5 | 5 | 4.21 | 953 | 55.1 | Mane-A019 Mane-A052 | Mane-B016 Mane-B089 | Engerix-B |
| Z14302 | SIV+ | M | 4.9 | 7.7 | 5 | 5 | 3.87 | 945 | 116.7 | Mane-A018  Mane-A082 | Mane-B015  Mane-B047 | DNA +Protein |
| Z15182 | SIV+ | M | 4.2 | 9.6 | 5 | 5 | 1.61 | 1029 | 62.7 | Mane-A009  Mane-A019 | U.D. | Engerix-B |
| Z15268 | SIV+ | M | 4.0 | 6.9 | 5 | 1 | 2.86 | 1030 | 68.0 | Mane-A006  Mane-A031 | Mane-B118  Mane-B120 | DNA +Protein |
| Z15374 | SIV+ | M | 3.6 | 6.2 | 5 | 1 | 3.38 | 681 | 53.4 | Mane-A006  Mane-A010 | Cannot Call | Engerix-B |
| Z15032 | SIV+ | M | 4.4 | 6.5 | 5 | 1 | 3.01 | 1046 | 37.8 | Mane-A006 Mane-A031 | Cannot Call | Engerix-B |
| Z15015 | Naïve | M | 5.1 | 9.5 | 25 | 5 | _ | N.D. | _ | Mane-A052  Mane-A084 | Cannot Call | None |
| Z15389 | Naïve | M | 4.3 | 7.6 | 25 | 5 | _ | N.D. | _ | Mane-A007b  Mane-A115 | Cannot Call | None |
| Z16293 | Naïve | M | 3.4 | 7.7 | 25 | 5 | _ | N.D. | _ | Mane-A032 | Cannot Call | None |
| Z16270 | Naïve | M | 4.0 | 5.0 | 5 | 5 | _ | N.D. | _ | N.D. | N.D. | None |
| Z18113 | Naïve | M | 2.1 | 3.6 | 5 | 5 | _ | N.D. | _ | N.D. | N.D. | None |
| Z17046 | Naïve | M | 3.6 | 4.0 | 5 | 5 | _ | N.D. | _ | Mane-A004  Mane-A019 | Mane-B118  Mane-B122 | None |

Abbreviations: ID, identification; i.m., intramuscular; M, male; N.D., not determined; U.D., undetermined

^a^ DNA and protein vaccine regimen comprised of HBV core and surface antigens and anti-CD180

| **Supplemental Table 2. Comparison of immunogenicity based on vaccine dose, number of sites, or vaccine history** | | | | |
| --- | --- | --- | --- | --- |
|  | Medians ± Interquartile Ranges | |  | *p*-value^a^ |
| Vaccine Dose | 25 μg  (n=6) | 5 μg  (n=9) |  |  |
| IFN-γ + T-cells^b^ | 25.0 ± 160 | 0 ± 69.8 |  | 0.096 |
| bAb^c^ | 15.4 ± 251 | 5.49 ± 21.7 |  | 0.145 |
| nAb^d^ | 25.0 ± 75.0 | 5.0 ± 15.0 |  | *0.049* |
| i.m. Sites | Multiple  (n=12) | SS  (n=3) |  |  |
| IFN-γ + T-cells^b^ | 0 ± 160 | 0 ± 27.08 |  | 0.664 |
| bAb | 6.5 ± 254 | 9.59 ± 20.7 |  | 0.945 |
| nAb | 5.0 ± 75.0 | 10.0 ± 15.0 |  | 0.664 |
| Pre-vaccination with  HBV Vaccine^e^ | DNA +Protein (n=4) | Engerix-B  (n=5) |  |  |
| IFN-γ + T-cells^b^ | 6.67 ± 69.8 | 0 ± 36.7 |  | 0.921 |
| bAb | 20.7 ± 251 | 4.65 ± 9.55 |  | 0.111 |
| nAb | 15.0 ± 75.0 | 5.0 ± 5.0 |  | 0.127 |

Abbreviations: bAb, binding antibody; i.m., intramuscular; nAb, neutralizing antibody; SS, single site

^a^ Mann-Whitney test was used to generate *p*-values; *p* < 0.05 is considered significant and indicated by italicized text.

^b^ IFN-γ SFC/10^6^ PBMCs

^c^ IgG μg/ml

^d^ PRNT_80_ reciprocal titer

^e^ SIV+ animals only; DNA and protein vaccine regimen comprised of HBV core and surface antigens and anti-CD180
